# Ethical and Philosophical Considerations for Gain-of-Function Policy: The Importance of Alternate Experiments

**DOI:** 10.3389/fbioe.2018.00011

**Published:** 2018-02-08

**Authors:** Nicholas Greig Evans

**Affiliations:** ^1^Department of Philosophy, University of Massachusetts Lowell, Lowell, MA, United States

**Keywords:** gain-of-function, biosecurity, dual-use research, biosafety, highly pathogenic avian influenza virus, coronavirus, bioethics

## Abstract

The Department of Health and Human Services Framework for Guiding Funding Decisions about Proposed Research Involving Enhanced Potential Pandemic Pathogens (PPPs) contains a series of principles for governing the funding and conduct of gain-of-function (GOF) research resulting in the creation of PPPs. In this article, I address one of these principles, governing the replacement of GOF research with alternate experiments. I argue that the principle fails to address the way that different experiments can promote the same values as those promoted by GOF research resulting in PPPs. I then address some objections to this claim, and provide policy recommendations moving forward.

Concerns over the accidental or deliberate release of novel pathogens has prompted a debate about the conduct or funding of biological research in the name of human health and security. Of particular concern is “gain-of-function” (GOF) research resulting in the creation of a “potential pandemic pathogen (PPP)” (GOF/PPP research) where, *inter alia*, the host range, virulence, or transmissibility of a pathogen is enhanced. For example, in 2011 researchers modified highly pathogenic avian influenza (HPAI) H5N1 to transmit via respiratory droplets in mammals (Herfst et al., 2012; Imai et al., 2012).

The 2011 HPAI H5N1 studies, and indeed all GOF/PPP research, generate *biosafety* and *biosecurity* concerns.^1^ Initial concern over the 2011 HPAI H5N1 studies focused on biosecurity: the risk of a deliberate release of an organism through, e.g., the use of a biological weapon (Evans, 2013). This concern evolved to encompass biosafety concerns that an accidental release of recombinant influenza could cause a global pandemic (Lipsitch and Galvani, 2014; Lipsitch and Inglesby, 2014; Evans et al., 2015).

In 2014, in response to these concerns, the US Government imposed a funding pause of GOF research involving influenza, severe acute respiratory syndrome (SARS) coronavirus, and Middle East respiratory syndrome (MERS) coronavirus (The White House, 2014). The pause was accompanied by a deliberative process undertaken by the US National Academies of Sciences, Engineering and Medicine (2016), National Research Council Institute of Medicine (2015), and National Science Advisory Board for Biosecurity (2016) to develop policy that managed the risks and benefits of GOF/PPP research. The resulting *Recommended Policy Guidance for Departmental Development of Review Mechanisms for Potential Pandemic Pathogen Care and Oversight (P3CO)* (Office of Science and Technology Policy, 2017), informed by the deliberative process, contained a set of principles that any GOF/PPP research ought to satisfy (Box 1), and associated guidance for managing the risks of GOF/PPP research once funded. The principles are largely drawn, without alteration, from the principles found in the *Recommendations for the Evaluation and Oversight of Proposed Gain-of-Function Research* (hereafter, *Recommendations)* published by the National Science Advisory Board for Biosecurity (2016). On December 19, 2017, these principles were reproduced in the Department of Health and Human Services’ (DHHS) *Framework for Guiding Funding Decisions about Proposed Research Involving Enhanced Potential Pandemic Pathogens*, marking an end to the funding pause.

Box 1 **Policy principles.**

Agency review mechanisms pursuant to this recommended policy guidance should establish that a project involving the creation, transfer, or use of enhanced PPPs should satisfy the following principles, which are based on similar principles in the NSABB.

Recommendations:

3.1. The proposal or plan for such a project has been evaluated by an independent expert review process (whether internal or external) and has been determined to be scientifically sound.
3.2. The pathogen that is anticipated to be generated by the project must be reasonably judged to be a credible source of a potential future human pandemic.
3.3. An assessment of the overall potential risks and benefits associated with the project determines that the potential risks as compared to the potential benefits to society are justified.
3.4. There are no feasible, equally efficacious alternative methods to address the same question in a manner that poses less risk than does the proposed approach.
3.5. The investigator and the institution where the project would be carried out have the demonstrated capacity and commitment to conduct it safely and securely and have the ability to respond rapidly, mitigate potential risks and take corrective actions in response to laboratory accidents, lapses in protocol and procedures, and potential security breaches.
3.6. The project’s results are anticipated to be responsibly communicated, in compliance with applicable laws, regulations, and policies, and any terms and conditions of funding, in order to realize their potential benefit.
3.7. The project will be supported through funding mechanisms that allow for appropriate management of risks and ongoing Federal and institutional oversight of all aspects of the research throughout the course of the project.
3.8. The project is ethically justifiable. Non-maleficence, beneficence, justice, respect for persons, scientific freedom, and responsible stewardship are among the ethical values that should be considered by a multidisciplinary review process making decisions about whether to fund research involving PPPs (Office of Science and Technology Policy, 2017).

A diagnostic for the success of this policy process, and indeed a mark of progress after 6 years of debate about GOF/PPP research^2^ would be to ask “does this policy adequately select between GOF/PPP research that on balance advances scientific knowledge and human values, and research that poses unacceptable risks to human health and security?” Here, I argue the answer to this question is “no.” The *P3CO* principles fail to select between GOF/PPP experiments by failing to consider that alternative experiments may provide a superior expected net benefit. In doing so, the policy fails to account for a range of important opportunities to enhance the safety and security of the life sciences without unduly burdening researchers or policymakers, or sacrificing meaningful progress in the life sciences.

In what follows, I describe the *P3CO* principles and their relationship to GOF/PPP research. I then argue that the principle suggesting that GOF/PPP research is justified if “no feasible, equally efficacious alternative methods to address the same question in a manner that poses less risk than does the proposed approach” is inappropriately permissive. I consider a series of objections to my account, and argue that none are sufficiently strong to justify the principle as it stands. I conclude with an alternate framing of these principles in the context of the DHHS policy.

## The Policy Principles

The *P3CO* principles echo the *Recommendations*’ guidance on federal department pre-funding review and approval of GOF/PPP research. These principles apply to “Gain of Function Research of Concern” (GOFROC): GOF research that is (1) highly transmissible and likely capable of wide and uncontrollable spread in human populations and (2) highly virulent and likely to cause significant morbidity and/or mortality in humans (National Science Advisory Board for Biosecurity, 2016). *Recommendations, P3CO*, and the DHHS policy note that all principles must be satisfied in order to pursue GOFROC. The document also suggests other risk mitigation strategies should biosafety and/or security issues arise post-funding, including measures should the final publication of results pose an information risk. I have discussed information risk posed by GOF/PPP research—of which I consider GOFROC a subset^3^—elsewhere (Evans, 2013; Evans and Selgelid, 2014), and set this aside for the purpose of my analysis.

*P3CO* and the final HHS policy outline the following principles for the funding and conduct of GOFROC. First, GOFROC must be subject to independent review to determine it answers a scientifically sound question. While the NSABB intimated in their articulation of this principle that this review should ideally be *supplemental* to existing review mechanisms for funded research, the *P3CO* principles remain silent on whether existing grant review mechanisms (which presumably select for scientifically sound projects) satisfy this principle.

Second, pathogens anticipated to be created by projects subject to the policy must be a credible source of a potential future human pandemic. Moreover, the experiments must also be assessed to provide benefits that outweigh the risks of conducting GOFROC. These principles respond to general concern that some GOF/PPP research presents risks that ultimately outweigh its potential benefits. The 2011 HPAI H5N1 papers, for example, were published after the NSABB reviewed revised drafts of the papers, which defended the public health benefits of the research to respond to an emerging HPAI H5N1 pandemic (Enserink, 2012). Interestingly, the language between *Recommendations* and *P3CO* changes significantly regarding the second principle above: the former requires that the pathogen be expected to “arise through natural processes,” while the latter merely requires a “credible” source, opening the possibility that GOFROC research might be funded to address human-engineered PPPs.

My analysis will focus on the fourth principle, which is that research should be funded only if “there is no feasible, equally efficacious alternative method to address the same question in a manner that poses less risk than does the proposed [GOFROC] approach.” The *Recommendations* add that

Alternative approaches must be explored and critically examined before funding [GOFROC]… modifications of the experimental design, use of attenuated or other strains that pose fewer risks to humans, or different approaches with less risk that may provide the same or very similar information may be feasible. Lines of experimentation that entail less risk should be pursued whenever possible (National Science Advisory Board for Biosecurity, 2016, 45).

The next three principles are procedural components on GOFROC funding. First, researchers and their institutions must have sufficient capacity to mitigate the risks of the research. While paradigm cases of GOF/PPP research occurred within large and expensive, high-containment laboratories, the increasing power of the life scientists mean that resource constraints on GOF/PPP research will ease in the long term. The research must also be communicated in compliance with applicable laws and regulations, and “supported through funding mechanisms that allow for appropriate management of risks and ongoing Federal and institutional oversight of all aspects of the research throughout the course of the project.” That is, the funding mechanism under which these projects are conducted ought to be flexible enough to accommodate changes to methodology, or additional resource requirements for risk mitigation.

Finally, the principles require the project be ethically justifiable, citing “values”^4^ such as non-maleficence, beneficence, justice, respect for persons, scientific freedom, and responsible stewardship. While *P3CO* does not articulate what these mean for the governance of GOFROC, the *Recommendations* reference an ethical white paper produced during the deliberative process (Selgelid, 2016).

## What’s in a Question? The Fourth Policy Principle’s Implications

A critical question is what these principles might permit or exclude. The NSABB acknowledged some GOF/PPP research (or GOFROC) might be too risky to fund or conduct, but did not give a specific example of what such an experiment might entail (National Science Advisory Board for Biosecurity, 2016, p. 4). Bearing in mind that GOFROC must meet all of these criteria some research is clearly excluded from funding: research that does not ask a scientifically sound question, does not address a credible source of human pandemic, or whose expected benefits are clearly outweighed by its expected risk. Moreover, in cases where a researcher or institution is unable to comply with federal laws and regulations, or ensure a safe research environment in the context of GOFROC, funding ought not be given for such experiments.

When it comes to the fourth principle—that the research cannot be feasibly or efficaciously pursued through another methodology that answers the same scientific question—it isn’t clear that any experiment would fail to satisfy this criterion. Methodologies in the life science closely track the organization of the field, its priorities, and the kinds of question that are asked. In a very simple sense, different methodologies, even if they create similar (or similarly valuable) knowledge, do not ask “the same question.” To change methodology is, in a very basic but important sense, to change the question.

This principle highlights, moreover, a strong division in the debate over GOF/PPP research. Advocates for GOF/PPP research have argued that GOF as a methodology has unique epistemic merits. While other experiments may allow us to demonstrate the *potential* for a pathogen to alter its host range or experience enhanced transmissibility or virulence, advocates maintain that only GOF can *show* us this is possible. As such, a change to an alternate methodology deprives us of the one methodological tool we have to conclusively prove that a (wild-type) virus can acquire the potential to cause a human pandemic (Casadevall et al., 2014).

It is thus difficult to understand, then, what work is done by the fourth principle as it is written. One suggested case in which an alternate methodology might answer the same question would be one in which a specific part of a pathogen’s structure altered to demonstrate a change in function.^5^ Say, for example, we wanted to determine whether a virus with a substituted HA protein would bind to human receptors. It would be possible to answer this question using an attenuated virus rather than its wild-type progenitor. A more radical alternative might be cell-free study of single proteins—for example, H5 or H7 receptor binding to mammalian sialic acids—could take the place of GOF/PPP research with similar aims (Lipsitch and Galvani, 2014) and eliminate the need for a live virus (at least, in initial research).^6^ This a rare example, however, and does not represent paradigm GOF/PPP studies studying which mutations in a viral genome are attributed to changes in phenotype under laboratory conditions. Alternate experiments to *these* GOF/PPP experiments may answer-related questions (Lipsitch and Galvani, 2014) and have considerable scientific and public health value, but do not answer precisely the same question. They are thus excluded as potential replacements for GOF/PPP studies under the new policy.

Yet the existence of alternate experiments, and the serious risks presented by some GOF/PPP research gives us a *pro tanto* justification for substituting experiments even if they do not ask the same question. This justification is particularly compelling if alternate experiments advance therapeutics development, disease surveillance, or public health response to emerging pandemics. Next, I’ll consider three objections that might arise from those who may wish to retain the current formulation of the fourth principle.

## Potential Objections

Here, I outline four potential objections and respond to them. These are:

1. Assert the P3CO principles are appropriate in the context of existing scientific governance;
2. Beyond instrumental benefits, scientists should be free to choose which questions they ask;
3. Appeal to the NSABB Recommendations for guidance on “same or similar” questions;
4. Appropriately tailoring the risk-benefit difference in principle three will select against the right set of experiments.

I respond to these in turn.

One could contend these *P3CO* principles are appropriate in the context of existing scientific governance. That is, as long as the expected risk-benefit difference is positive (i.e., sufficiently in favor of benefit), the (scientifically meritorious) research ought to proceed. We ought not be in the business of guessing whether other questions might give equally valuable answers, or trying as policymakers to guess which scientific question is more worthy of pursuing.

Advocates typically justify GOF/PPP experiments on their role in achieving some other end, e.g., developing novel vaccines or therapeutics (Schultz-Cherry et al., 2014), or enhancing disease surveillance (Casadevall et al., 2014). There is some value in GOF/PPP research for its own sake, but this is the case for any scientifically meritorious question. Moreover, the value of preventing an accidental or deliberately caused disease pandemic arguably outweighs the mere value of scientific knowledge for its own sake. Advocates of GOF/PPP don’t just leverage the instrumental value of these studies in their argument; they rely on them.

Yet funders prioritize those ends, and thus partly determine what scientific questions can be asked; we already do what advocates of GOF/PPP research deny. Allowing similar, but not identical questions to take the place of GOF/PPP research amounts a small change in degree from existing practice. Only occasionally is GOF/PPP research a unique means to medical and public health ends (Evans, 2014); other experiments may answer different scientific questions, but *still answer questions that achieve the same ends as GOF/PPP research*. The GOF benefit analysis conducted during the deliberative process demonstrated that only in 9 of 24 scientific and public health goals addressed for influenza was GOF/PPP uniquely useful; this number was only 3 in 13 for coronavirus (SARS and MERS) research (Gryphon Scientific, 2016, pp. 249–254).

A proponent of GOF/PPP research might reply that what matters isn’t (only) that we achieve our scientific or public health aims, but that scientists are also free to choose which questions they ask. Scientific freedom, after all, is not simply an efficient means to achieve material ends, but valuable for its own sake (Evans, 2013). This response also fails: scientific freedom is important, but we already acknowledge limits on that freedom given certain risks to others (e.g., in human experimentation). Given that the set of experiments we can permissibly fund—and thus, the questions we ask—is already much smaller than the total set of scientific questions we have available to ask, it seems minimally invasive to ask that where possible, we pick from a set of related questions that accomplish our aims while entailing fewer, or less extreme, risks.

One might appeal to the *Recommendations* for guidance. In their fourth recommendation, the NSABB notes that review of GOFROC should include consideration of alternate methodologies that provide the “same or very similar information” at lower risk. This presumably a larger set than methodologies that strictly answer the same scientific question. How large a set this is, however, depends on what constitutes “information” in this sense, and what our basis for comparison determine similarity might be.

What might this conception of similarity look like? Consider an interesting piece of scientific knowledge such as “the sequence changes required to confer mammalian transmissibility via respiratory droplets onto HPAI viruses (Watanabe et al., 2014).” GOF/PPP experiments, at least those that spurred the deliberative processes, ultimately provided one set of information that produces the kind of scientific knowledge we want. It did so, moreover, in a very specific way: by providing the exact sequence change required for specific isolates of HPAI. In the case of the 2011 experiments, these were sequence changes for influenza virus A/Indonesia/5/2005 (Herfst et al., 2012), and six reassortant viruses from a library of reassortants from A/Vietnam/1203/2004 (H5N1) and A/Puerto Rico/8/34 (H1N1).

Critics have noted that while these experiments provide sequence changes required to confer mammalian transmissibility via respiratory droplets onto HPAI viruses, the sequence changes developed in laboratories may not be likely or even plausible in wild flu viruses (Enserink, 2011; Lipsitch and Galvani, 2014). Other methodologies may provide likely sequence changes for a broader range of viruses, some (or more) of which are plausible in nature. This provides a similar kind of knowledge, albeit less precise (in the sense that it does not provide us with an exact sequence) but with a greater degree of generalization. Both give us our sought after knowledge in some sense of the initial parameters.

How we understand similarity depends, in part, on what kinds of knowledge we find valuable. Let us assume, plausibly, that the general scientific interest of each study is equal (though perhaps to different sets of scientists). Whether these two sets of experiment provide relevantly “very similar” information depends, again, on the relationship between our ends and the kinds of knowledge about which we are interested. If our interest is cataloging individual HPAI viruses for their capacity to undergo sustained transmission and are able to look for those specific sequences in nature, then GOF/PPP experiments occupy a fairly narrow space in which we could find other, similar experiments that generate similar information. If, however, we are interested in determining which kinds of sequence are most likely to appear in flu viruses in the wild, GOF/PPP experiments are arguably not unique and may even by counterproductive as a form of knowledge generation (Lipsitch, 2014). How we define similarity depends on our ends; *P3CO*’s reduction from similarity to *identity* commits us to valuing scientific information in a way we might otherwise not endorse.

While I have used the 2011 HPAI H5N1 studies in my argument, other experiments may have different goals for which GOF/PPP methodologies are more suited. Studies involving MERS coronavirus, for example, were allowed to continue after the initial pause because their aim was to develop an animal model for MERS in which to conduct experiments (Kaiser, 2014). Arguably, GOF/PPP research in aid of this pursuit may contribute uniquely to the development of an animal model in MERS or be so efficacious a method as to outweigh potential risks.

Finally, one might respond that the most crucial aspect here is the risk-benefit difference in principle three. That is, one might argue that the primary principle on which we ought to judge GOF/PPP research is whether its expected benefits outweigh its expected risks. Moreover, if we set our threshold for acceptable risk correctly, we will exclude those experiments that are too risky relative to their expected benefits and default to other methodologies.

This is promising insofar as it forces us to specify exactly how much our purported benefits must exceed potential risks in order to justify funding GOF/PPP experiments. Yet it elides the *comparative* nature of risk assessment. Decisions under conditions of risk are, and ought to be, comparative (see, e.g., Hansson, 2003). Say we believe the right decision is one that maximizes the probability-weighted net benefits of our actions. And let us also say a GOF/PPP experiment might lead to the development of a novel vaccine: say, a 90% chance, and would save 10 million people; but also entails an independent nontrivial “global catastrophic risk” of 0.01% of a disease pandemic that kills 1 billion people. Our probability adjusted net benefit is thus 8.9 million people: we expect, all things considered, that many more people will be saved than die as a result of our experiment. Now imagine an alternate experiment called alt-GOF. This experiment has the same probability of causing benefit or harm, but the magnitude of the benefit and harms are 9.9 million and 10 million, respectively. Our expected value is now 8.909 million lives: about 9,090 more than the initial case. It isn’t sufficient to say GOF leads to a positive net benefit. We have clear reason to prefer alt-GOF to GOF/PPP, regardless of whether or not it answers the exact same question.^7^

Risk assessments ought to be understood as comparisons rather than absolute references (National Research Council Institute of Medicine, 2015). It isn’t sufficient to say we are justified in funding GOF/PPP research just in case its expected benefits outweigh its expected risks. Rather, we want to say GOF/PPP provides the *most* justifiable tradeoff between benefit and risk. What that tradeoff ought to be is unclear from the *P3CO* policy. Regardless of what criteria we use for decision-making under risk, our principles for governance ought to be comparative. Principle four undercuts this by precluding a large set of options from which comparison can follow.

## Recommendations

In response to my argument, the following solutions could be implemented individually or in concert.

(1) Clarify principle four in broader terms. HHS and other agencies could release additional guidance that would clarify principle four to include considerations of a reasonable range of alternative experiments with independent scientific merit that promote the same aims as GOF/PPP research. A move away from the “same question” or “very similar information” to a broader conception of alternatives would develop the governance of this research in important ways, critically in forcing policymakers and scientists to specify what, exactly, they wish GOF/PPP research to accomplish.
  This is a plausible in the context of section V of the current HHS policy, which claims that “HHS will periodically re-evaluate and modify this review process, as necessary, to reflect scientific advances and changes to the regulatory landscape” (Department of Health and Human Services, 2017). We could envisage such a review tackling, first, the question of alternate experiments and building on existing work [e.g., the risk and benefit assessment from the deliberative process (Gryphon Scientific, 2016)] to develop specific guidance on alternate experiments that fulfill a range of scientific and public health aims.
(2) Introduce comparative subprinciples into principle three. That is, rather than attempt to realign principle four with the above critiques, further incorporate comparative risk assessment into the third principle and its translation into actionable policy. By explicitly noting that risk assessments must be comparative over a range of plausible alternatives within the broader aims of the funded research, agencies can side-step potential issues with principle four.
  This recommendation would be a plausible alternative to my first recommendation by incorporating the substance of my critique into principle three, rather than principle four. It would conceivably render principle four unnecessary. Rather, the risk assessment would be comparative and account for alternatives that produce valuable scientific information that is both similar to and as *valuable* as proposed GOF/PPP research, but may entail fewer serious risks. This could be accomplished without a specific policy change in the context of the HHS policy, instead built into future procedural elements of the review process that have yet to be described. The risk here is that excluding principle four as an explicit consideration reopens the possibility that alternates are never taken seriously; I acknowledge this, but note that my above suggestion, taken in good faith, makes this unlikely.
(3) Fund comparative assessments of scientific research and alternatives to GOF/PPP. Requiring a risk and benefit analysis as part of policy is itself challenging, and other policies on dual-use research of concern have been criticized by practitioners for requiring analysis that is beyond the skills of any institutional oversight body. Rather than require *de novo* analysis for each GOF/PPP experiment to arise, research on these experiments—using the risk and benefit analysis from the deliberative process as a template—could develop a hierarchy of comparatively low-, medium-, and high-risk experiments, and the conditions under which they are uniquely justified or overwhelmingly efficacious. A kind of “GOF/PPP case law” is itself a valuable addition to the life sciences, and an important policy innovation.

We already have some precedent for this in the form of those MERS studies that were reinstated after the initial pause. This kind of research arguably provides us with an important data point on what counts as efficacious and justified GOF/PPP research looks like. In contrast, research that created mammalian transmissible variants of a virus that has never been recorded in mammals (Sutton et al., 2014) is arguably a high-risk, unjustified form of GOF/PPP research.

The new GOF/PPP principles require further interpretation to become effective governance. In this article, I have argued that principle four, “the research cannot be feasibly or efficaciously pursued through another methodology that answers the same scientific question,” is overly permissive and have suggested reform for policymakers. The debate over GOF/PPP research is surely not over, and whether these changes are ultimately incorporated is a matter for future policy work.

## Author Contributions

NE responsible for all research and writing.

## Conflict of Interest Statement

The author declares that the research was conducted in the absence of any commercial or financial relationships that could be construed as a potential conflict of interest.

## Footnotes

^1^These definitions vary, e.g., some use one term to refer to both accidental and intentionally caused outbreaks; or using a third term to refer to the inclusive disjunction of biosafety and biosecurity as defined above. The World Health Organization, in particular, uses biosafety and biosecurity in similar ways to those I use here (i.e., biosafety *qua* accidental, biosecurity *qua* intentional theft, or misuse), and uses “biorisk” to encompass both (World Health Organization, 2006). I note these alternatives for any reader attempting to match the concepts across the literature.

^2^And 16 years of debate about “dual-use research,” which encompasses but is not limited to GOF/PPP research (Evans, 2013; Evans et al., 2015).

^3^In general, I use GOFROC when explicitly referring to the policy debate, but GOF/PPP as the larger set of experiments to which my general argument applies. There may be other research that is GOF/PPP research, and should be regulated in the same way, but is not GOFROC.

^4^I place values in quotes because it isn’t immediately clear what kind of values these are. The first four are drawn from Beauchamp and Childress, who articulate them as mid-level principles that point at a range of fundamental values, but do not depend on our strict commitment to particular values for us to agree to their importance (Beauchamp and Childress, 2012). For example, respect for persons is derived from a (primarily negative) conception of autonomy, that is, being able to pursuing one’s own projects without interference by others. Scientific freedom, on the other hand, need not be a “value” per se, but may be *valuable* as a component of the right to freedom of speech (Evans, 2013) or freedom of inquiry (Miller and Selgelid, 2008). I won’t inquire more into these ethical principles, except to note that their status as values is subject to considerable debate.

^5^I’m indebted to Corey Meyer for suggesting this alternate.

^6^Thanks to an anonymous reviewer for this suggestion.

^7^This sets aside we should apply expected value calculations to statistically unlikely but highly impactful consequences—like a catastrophic risk (see, e.g., Lipsitch et al., 2016).
